# Widespread presence of "bacterial-like" PPP phosphatases in eukaryotes

**DOI:** 10.1186/1471-2148-4-47

**Published:** 2004-11-19

**Authors:** Alexandra V Andreeva, Mikhail A Kutuzov

**Affiliations:** 1 Research School of Biological and Molecular Sciences, Oxford Brookes University, Headington, Oxford OX3 OBP, UK; 2 Present address: University of Illinois, College of Medicine, Department of Pharmacology, 835 S. Wolcott Ave, Chicago, IL 60612, USA

## Abstract

**Background:**

In eukaryotes, PPP (protein phosphatase P) family is one of the two known protein phosphatase families specific for Ser and Thr. The role of PPP phosphatases in multiple signaling pathways in eukaryotic cell has been extensively studied. Unlike eukaryotic PPP phosphatases, bacterial members of the family have broad substrate specificity or may even be Tyr-specific. Moreover, one group of bacterial PPPs are diadenosine tetraphosphatases, indicating that bacterial PPP phosphatases may not necessarily function as protein phosphatases.

**Results:**

We describe the presence in eukaryotes of three groups of expressed genes encoding "non-conventional" phosphatases of the PPP family. These enzymes are more closely related to bacterial PPP phosphatases than to the known eukaryotic members of the family. One group, found exclusively in land plants, is most closely related to PPP phosphatases from some α-*Proteobacteria*, including *Rhizobiales*, *Rhodobacterales and Rhodospirillaceae*. This group is therefore termed *Rhizobiales */ *Rhodobacterales */ *Rhodospirillaceae*-like phosphatases, or Rhilphs. Phosphatases of the other group are found in *Viridiplantae*, *Rhodophyta*, *Trypanosomatidae*, *Plasmodium *and some fungi. They are structurally related to phosphatases from psychrophilic bacteria *Shewanella *and *Colwellia*, and are termed *Shewanella*-like phosphatases, or Shelphs. Phosphatases of the third group are distantly related to ApaH, bacterial diadenosine tetraphosphatases, and are termed ApaH-like phosphatases, or Alphs. Patchy distribution of Alphs in animals, plants, fungi, diatoms and kinetoplasts suggests that these phosphatases were present in the common ancestor of eukaryotes but were independently lost in many lineages. Rhilphs, Shelphs and Alphs form PPP clades, as divergent from "conventional" eukaryotic PPP phosphatases as they are from each other and from major bacterial clades. In addition, comparison of primary structures revealed a previously unrecognised (I/L/V)D(S/T)G motif, conserved in all bacterial and "bacterial-like" eukaryotic PPPs, but not in "conventional" eukaryotic and archaeal PPPs.

**Conclusions:**

Our findings demonstrate that many eukaryotes possess diverse "bacterial-like" PPP phosphatases, the enzymatic characteristics, physiological roles and precise evolutionary history of which have yet to be determined.

## Background

Reversible phosphorylation of proteins is a ubiquitous mechanism, indispensable for regulation of virtually any cellular function. Therefore, protein kinases and phosphatases are of paramount importance for normal functioning of all metabolic and signalling pathways. In eukaryotes, PPP family is one of the two known protein phosphatase families specific for Ser and Thr [1-4]. Unlike eukaryotic (and archaeal [5]) PPP phosphatases, bacterial members of the family have broad substrate specificity [6] or may even be Tyr-specific [7-9]. Moreover, one group of bacterial PPPs are diadenosine tetraphosphatases [10,11]. Unlike eukaryotes, in prokaryotes PPP phosphatases appear to be facultative, since entirely sequenced genomes of some bacteria and archaea do not encode them [5,12]. Nevertheless, when present, they appear to play essential roles [13-15].

Three motifs (GDXHG, GDXXDRG and GNH(E/D)), highly conserved in the N-terminal subdomains of the catalytic domains of all PPP phosphatases [10,11], contain most residues which coordinate metal ions in the active centre [16] and are considered as the signature of the PPP family.

In a previous work [17] we identified an unusual cDNA fragment from a moss *Physcomitrella patens*, showing no similarities to the known PPP phosphatases beyond the presence of the GDXHG and GDXXDRG motifs. Detection of homologous cDNA sequences from *Arabidopsis *and rice suggested the presence of an unknown PPP group in plants, distinct from "conventional" eukaryotic PPP phosphatases [17].

We have now taken advantage of a much greater representation (as compared to 1999) of sequence databases for various species to further explore this initial observation. We present the evidence for the existence in eukaryotes of three "non-conventional" branches of the PPP family. We also identify a previously unrecognised conserved motif in the PPP catalytic domain, which can be used as a signature of "bacterial"-type PPP phosphatases.

## Results

### "Rhizobiales / Rhodobacterales / Rhodospirillaceae – like" PPP phosphatases in plants

**Figure 1:**
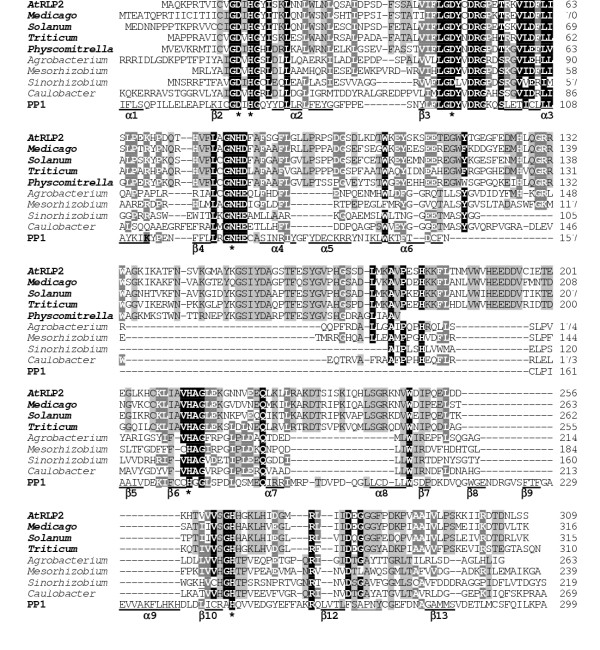
**Comparison of the primary structures of plant Rhilphs, related α-proteobacterial phosphatases and human PP1α as a prototype of "conventional" eukaryotic PPP phosphatases**. Amino acid residues conserved in at least all but one Rhilphs and α-proteobacterial phosphatases are shown in white and shaded in black. Residues conserved in at least two thirds of the sequences are shown in white and shaded in dark grey. Residues conserved in at least half of the sequences are shown in black and shaded in light grey. Following substitutions were considered as conserved residues: Ile/Leu, Phe/Tyr, Asp/Glu, Asn/Gln, Arg/Lys and Ser/Thr. Catalytic site residues that interact with metal ions are indicated by asterisks according to [20]. SAPNY motif in PP1, conserved in most eukaryotic PPP phosphatases, is double underlined. *Solanum tuberosum *sequence is translation of the EST entries BQ516856, BQ516857 and BI435517. *Physcomitrella patens *sequence is translation of the EST entry BQ039171. Other accession numbers are indicated in Table 1.

Two *Arabidopsis *sequences, At3g09960 and At3g09970, were retrieved using the *P. patens *fragment [17] as a query in TBlastN searches. They share 85% identity with each other at the protein level (see Figure 1). Both genes are transcribed, since full-length cDNAs have been detected in a large-scale transcription study [18]. They are arranged on chromosome 3 in tandem, suggesting their origin by a recent duplication. A number of ESTs from other plant species (but none from non-plant eukaryotes) were also detected by TBlastN searches, which in most cases provide no evidence for the existence of more than one isoform.

Among prokaryotes, related sequences were detected in some α*-Proteobacteria*, the closest matches being with *Rhizobiales*, *Rhodobacterales and Rhodospirillaceae*. Therefore, we designate this group as *Rhizobiales */ *Rhodobacterales */ *Rhodospirillaceae*-like phosphatases, or Rhilphs (Figure 1; see also Table 1 and Figure 4), and the two *Arabidopsis *genes products At3g09960 and At3g09970 as "*Rhizobiales*-like" phosphatases 1 (RLP1) and 2 (RLP2), respectively.

**Table 1:** Species, accession numbers (UniProt, EMBL, NCBI or TIGR Gene Index) and common names (where available) of PPP phosphatase sequences shown in Figure 4. For *A. thaliana *sequences, gene numbers are also indicated. Sequence No. 67 is available from *Chlamydomonas reinhardtii *draft genome [65].

No.	Accession	Species name
**Rhilphs**		
1	Q9SR61; At3g09960	*Arabidopsis thaliana*
2	Q9SR62; At3g09970	*Arabidopsis thaliana*
3	BE034080	*Mesembryanthemum crystallinum*
4	BQ995369	*Lactuca sativa*
5	AW034786	*Lycopersicon esculentum*
6	BU875146	*Populus balsamifera*
7	BG457803	*Medicago truncatula*
8	AV425727	*Lotus japonicus*
9	BM731295	*Glycine max*
10	BF261816	*Hordeum vulgare*
11	BQ788728	*Triticum aestivum*
12	AL731641	*Oryza sativa*
13	CF670562	*Pinus taeda*
14	BQ039171	*Physcomitrella patens*
**Group I (α-Proteobacteria)**		
15	Q987U4	*Mesorhizobium loti*
16	Q8UA33	*Agrobacterium tumefaciens*
17	ZP_00054691	*Magnetospirillum magnetotacticum*
18	ZP_00015226	*Rhodospirillum rubrum*
19	ZP_00014771	*Rhodospirillum rubrum*
20	CAE28794	*Rhodopseudomonas palustris*
21	Q9ABQ8	*Caulobacter crescentus*
22	ZP_00051041	*Magnetospirillum magnetotacticum*
23	ZP_00093979	*Novosphingobium aromaticivorans*
24	Q92V37	*Sinorhizobium meliloti*
**Group VII (heterogeneous)**		
25	Q8YZT4	*Anabaena sp.*
26	Q9WZK1	*Thermotoga maritima*
27	O34205	*Fervidobacterium islandicum*
28	NZ_AABE01000101	*Cytophaga hutchinsonii*
**Group III (γ-Proteobacteria and bacteriophage λ)**		
29	P03772	Bacteriophage λ
30	P55798	*E. coli *PrpA
31	Q8VPE2	*Salmonella typhimurium *PrpA
32	Q8Z487	*Salmonella enterica*
33	P55799	*E. coli *PrpB
**Group IV (Firmicutes)**		
34	Q81YR3	*Bacillus anthracis*
35	Q9FB69	*Lactococcus lactis*
36	Q97FF3	*Clostridium acetobutylicum*
**Alphs**		
37	BM291808	*Amblyomma variegatum*
38	TC9835	*Ciona intestinalis*
39	BU652795	*Chlamydomonas reinhardtii*
40	AC091781	*Trypanosoma brucei*
41	AC084046	*Trypanosoma brucei*
42	AL499620	*Leishmania major*
43	BQ143558	*Metarhizium anisopliae*
44	AC127427	*Magnaporthe grisea*
45	AA966318	*Aspergillus nidulans*
46	P40152	*Saccharomyces cerevisiae*
**ApaH**		
47	Q8Y1K9	*Ralstonia solanacearum *ApaH
48	Q9JVF4	*Neisseria meningitidis *ApaH
49	P05637	*Escherichia coli *ApaH
**Group VI (heterogeneous)**		
50	O31614	*Bacillus subtilis*
51	O69213	*Anabaena sp. *PrpA
52	Q93JF4	*Streptomyces coelicolor*
53	Q9RS78	*Deinococcus radiodurans*
**Group II (Cyanobacteria)**		
54	O54390	*Microcystis aeruginosa *PP1-Cyano 1
55	P74150	*Synechocystis sp.*
56	Q8YP31	*Anabaena sp.*
57	ZP_00072257	*Trichodesmium erythraeum*
58	Q8DGA2	*Thermosynechococcus elongatus*
**Shelphs**		
59	AC119500*	*Leishmania major*
60	Q8EBN0	*Shewanella oneidensis*
61	Q9S427	*Shewanella sp.*
62	TIGR_167879 Contig1731	*Colwellia psychrerythraea*
63	CF394707	*Pinus taeda*
64	TC31593	*Solanum tuberosum*
65	Q944L7; At1g18480	*Arabidopsis thaliana*
66	BF645180	*Medicago truncatula*
67	Scaffold_45	*Chlamydomonas reinhardtii*
68	Q9LMJ5; At1g07010	*Arabidopsis thaliana*
69	AW266595	*Mesembryanthemum crystallinum*
70	BG644111	*Lycopersicon esculentum*
71	BG450922	*Medicago truncatula*
72	BI787505	*Glycine max*
73	Q8L676	*Oryza sativa*
74	TC21958	*Hordeum vulgare*
75	AC007863	*Trypanosoma brucei*
76	AL499621	*Leishmania major*
77	TIGR_246197 Contig433	*Myxococcus xanthus*
78	EAK84303	*Ustilago maydis*
79	O74480	*Schizosaccharomyces pombe*
80	EAK87480	*Cryptosporidium parvum*
81	Q7RIH8	*Plasmodium yoelii*
82	Q8IKE5	*Plasmodium falciparum*
83	Q8I5Y5	*Plasmodium falciparum*
84	Q7RR22	*Plasmodium yoelii*
**Group V (heterogeneous)**		
85	O87639	*Streptomyces coelicolor*
86	Q9RVT7	*Deinococcus radiodurans*
**Archaea**		
87	O28453	*Archaeoglobus fulgidus *PPA
88	Q8ZW26	*Pyrobaculum aerophilum*
89	O34200	*Methanosarcina thermophila *PP1-arch2
90	Y12396	*Pyrodictium abyssi*
**"Conventional" eukaryotic PPP**		
91	Q9U493	*Plasmodium falciparum *PPJ
92	Q8I728	*Trypanosoma cruzi *PPEF
93	BH900132	*Ostreococcus tauri*
94	O14829	*Homo sapiens *PPEF1 (PP7)
95	Q8IDE7	*Plasmodium falciparum *PP5
96	P53041	*Homo sapiens *PP5
97	P53043	*Saccharomyces cerevisiae *PPT
98	P32838	*Saccharomyces cerevisiae *PPG
99	P05323	*Homo sapiens *PP2A
100	O00743	*Homo sapiens *PP6
101	Q08209	*Homo sapiens *Calcineurin (PP2B)
102	P08129	*Homo sapiens *PP1
103	P32945	*Saccharomyces cerevisiae *PPQ
104	O49346	*Arabidopsis thaliana *PP7

* This phosphatase is unlikely to be catalytically active due to replacements of essential residues in the active centre.

**Figure 2:**
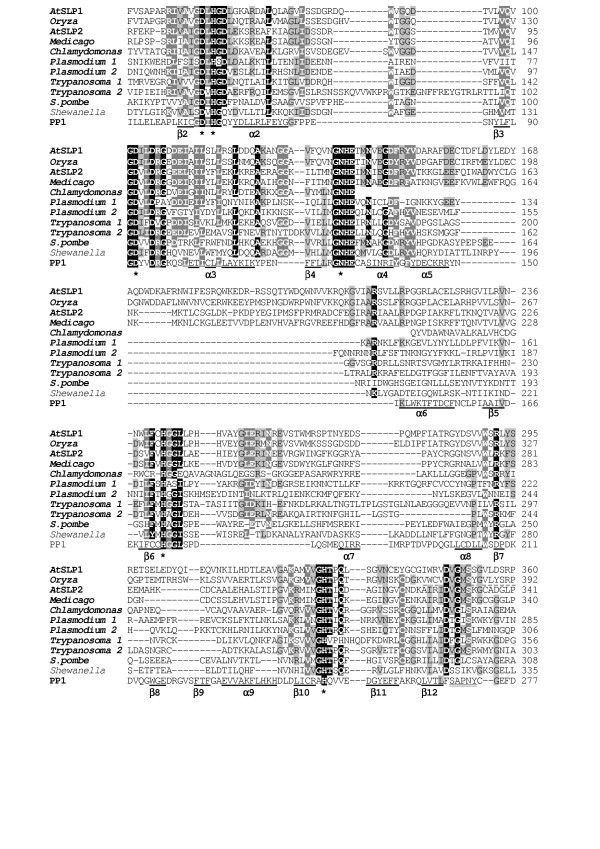
**Comparison of the primary structures of "*Shewanella*-like" phosphatases (Shelphs) and human PP1α as a prototype of eukaryotic PPP phosphatases**. Designations for conserved amino acid residues are as in Figure 1. For *Oryza sativa*, dashed underlined C-terminal sequence has been corrected by comparison with ESTs. Accession numbers: *Plasmodium falciparum *1, Q8I5Y5; 2, Q8IKE5; *Trypanosoma brucei *1, AC007863; 2, AC084046.12. *Chlamydomonas reinhardtii *sequence is translation of the EST entries BG855683 and BI995255. Other accession numbers are indicated in Table 1.

**Figure 3:**
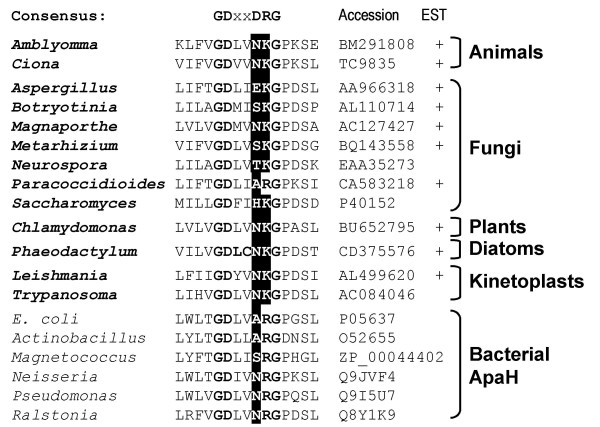
**Characteristic modifications (shaded in black) in the conserved PPP signature motif GDXXDRG in bacterial diadenosine tetraphosphatases and eukaryotic Alphs**. Eukaryotic species are shown in bold. Plus signs indicate that gene expression is confirmed by the presence of ESTs.

**Figure 4:**
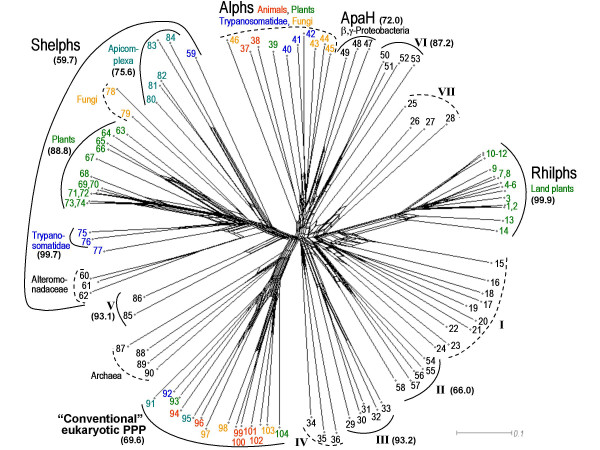
**Neighbor-Net analysis of the conserved N-terminal subdomains (starting 5 amino acid residues before conserved GDXHG and ending 25 residues after GNH(E/D) of 104 bacterial, archaeal and eukaryotic PPP phosphatases**. Bootstrap values exceeding 50% (out of 1000 resamplings) were obtained in a separate neihbour-joining analysis and are shown in brackets. Species and accession numbers are listed in Table 1. Note that groups designated as I, IV and VII did not receive significant bootstrap support; corresponding sequences are grouped together for convenience of their representation in Table 1. This image (and bootstrap values for the alternative splits) can be viewed at a higher resolution as the Additional File 1.

**Figure 5:**
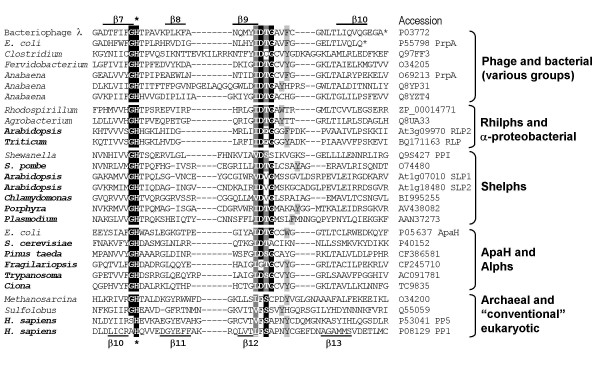
**Distinct conserved motifs in the C-termini of bacterial and "bacterial-like" PPP phosphatases from eukaryotes as opposed to archaeal and eukaryotic PPP phosphatases**. A His residue directly binding a metal ion in the catalytic centre (marked with asterisk), and the elements of secondary structure are shown for bacteriophage λ phosphatase and for human PP1 according to ref. [20] and [19], respectively. The (I/L/V)D(S/T)G motif, highly conserved in bacterial and "bacterial-like" phosphatases, is highlighted. An expanded version of this alignment can be viewed as the Additional File 2.

#### Structural features of Rhilphs

All residues that are expected to bind metal ions in the catalytic centre are conserved in Rhilphs (Figure 1). Rhilphs do not have N- or C-terminal extensions beyond their catalytic domains, which in many PPP phosphatases have regulatory function and / or interact with regulatory proteins / subunits. Instead, they have characteristic inserts between the conserved motifs GNH(E/D) and HAG (corresponding to HGG in "conventional" eukaryotic PPPs, see Figure 1). Notably, much shorter inserts are found at a similar position in α-proteobacterial phosphatases (group I in Figure 1). Inserts in both plant Rhilphs and α-proteobacterial phosphatases contain a conserved motif LXXAXPXXH (Figure 1). Similarly to bacteriophage λphosphatase (λPP [19]), Rhilphs lack a region corresponding to β8, β9 and α9 of eukaryotic PPPs [20-22]. Like bacterial PPP phosphatases, Rhilphs do not have a SAPNY motif, conserved in the β12-β13 loop of eukaryotic PPPs. Analysis of Rhilph sequences did not reveal targeting or signal peptides.

While this work was in progress, a phosphatase encoded by an *Arabidopsis *gene *At1g07010 *was reported in an independent study [4].

### "*Shewanella*-like" PPP phosphatases in plants, red algae, fungi and unicellular parasites

We undertook further TBlastN searches using full-length *Arabidopsis *RLP2 as a query to see whether *Arabidopsis *genome encodes additional "bacterial-like" PPPs. These searches identified two more genes for putative PPP phosphatases, only distantly related to Rhilphs and to any other members of the family (Figure 2).

One of these genes is *At1g07010*^1^. At least three different predicted products of this gene could be found in protein databases. On the basis of comparison with EST sequences, we consider as the correct structure that of Q8RY10 with Asp and Gly at positions 109 and 208, respectively (see Figure 2). The other detected gene, *At1g18480*, is also represented in the databases by three distinct deduced proteins. Comparison with *A. thaliana *ESTs confirms Q944L7 as the correct structure.

Genomic and EST database searches provided ample evidence for the presence of related phosphatases in a number of green plants, including multiple angiosperm species, pine and a unicellular green alga *Chlamydomonas reinhardtii *(Figure 2; see also Table 1 and Figure 4). Related sequences were also identified in some fungi (several basidiomycetes and an ascomycete *Schizosaccharomyces pombe*, but not other ascomycetes), in *Apicomplexa, Trypanosomatidae*, and in a red alga *Porphyra yezoensis *(for available sequence from the latter species, see Figure 5).

The most closely related prokaryotic phosphatases were detected in *Myxococcus xanthus *(δ-*Proteobacteria*) and psychrophilic bacteria *Alteromonadales *(γ-*Proteobacteria*): uncharacterised phosphatases from *Shewanella oneidensis *and *Colwellia psychrerythraea *and a Tyr-specific phosphatase PPI from *Shewanella sp*. [8]. Therefore, we designate this phosphatase group as "*Shewanella*-like" phosphatases, or Shelphs, and the products of the two prototype *Arabidopsis *genes At1g07010 and At1g18480 as "*Shewanella*-like" phosphatases 1 (SLP1) and 2 (SLP2), respectively.

#### Structural features of Shelphs

Like in Rhilphs, all residues that are expected to bind metal ions in the catalytic centre are conserved in Shelphs (see Figure 1). Another feature common with Rhilphs is the presence of inserts (as compared to "conventional" eukaryotic PPPs) between the GNHE and H(A/G)G motifs (Figure 1), which are especially long in plant Shelphs. However, these inserts share no sequence similarity between Rhilphs and Shelphs and probably appeared in the two phosphatase groups independently. Like in Rhilphs, a region corresponding to β8, β9 and α9 of eukaryotic PPPs is absent in Shelphs, and the primary structure of the region corresponding to the β12-β13 loop is similar to that of typical bacterial PPPs. *A.thaliana *SLP1 and corresponding Shelph isoform from rice have chloroplast targeting sequence, which could not be detected in *A.thaliana *SLP2 and corresponding isoform from *Medicago truncatula*.

### Eukaryotic PPPs distantly related to bacterial diadenosine tetraphosphatases

Identification of Rhilphs and Shelphs prompted us to perform extensive searches of eukaryotic sequence databases. These searches revealed the existence of other "bacterial-like" PPP phosphatases throughout eukaryotes. Sequences only distantly related to Rhilphs, Shelphs or any other PPP phosphatases were detected in several fungi (including a putative *S. cerevisiae *phosphatase reported previously [23]), in *Trypanosomatidae*, a tick *Amblyomma*, an ascidian *Ciona*, *Chlamydomonas*, pine and diatoms *Fragilariopsis cylindrus *and *Phaeodactylum tricornutum*. Blast searches using these sequences revealed that all of them share higher similarity to bacterial diadenosine tetraphosphatases (ApaH) than to other PPP groups. Therefore, we tentatively designate them as ApaH-like phosphatases, or Alphs (Figures 3 and 4; Table 1; partial sequences available for pine and diatoms are shown in Figure 5).

Alphs share a distinctive common structural feature. In the GDXXDRG motif, absolutely conserved in other PPPs, the second Asp (which stabilises the protonation of a His that directly participates in catalysis [20]) is replaced by a neutral amino acid, and the Arg residue (which coordinates phosphate [24]) is replaced, with one exception, by Lys (Figure 3). The former of these replacements is also found in ApaH, while the latter is unique to Alphs. While higher overall sequence similarity and a common alteration in the GDXXDRG motif are compatible with closer relatedness of Alphs to bacterial diadenosine tetraphosphatases, phylogenetic analysis using full length sequences failed to produce a robust tree due to high sequence diversity (not shown).

### Relationship of novel eukaryotic PPP groups to known PPP phosphatases

In order to better understand the relationship of "bacterial-like" PPP phosphatases in eukaryotes to each other and to bacterial PPPs, we attempted to extend our previous phylogenetic analysis of eukaryotic PPP phosphatases [25] by including PPP sequences from a number of bacteria and archaea. Primary structures of bacterial PPP phosphatases are extremely diverse and, outside the relatively conserved N-terminal subdomain of about 100 amino acids containing the GDXHG, GDXXDRG and GNH(E/D) motifs, they share only a few conserved residues. Moreover, many of the sequences have long insertions at different positions. This leads to the failure to produce informative alignments of full-length catalytic domains. Therefore, we aligned more conserved N-terminal subdomains only, an approach applied previously by Kennelly [6] to a much smaller set of PPP sequences available at that time. Phylogenetic reconstruction was attempted with either neighbor-joining (as implemented in PHYLIP [26] or SplitsTree [27]) or maximum likelihood analysis using quartet puzzling (TreePuzzle [28]); in the latter case a smaller dataset consisting of with consisting of 32 representative sequences was analyzed due to the inability of the algorithm to handle large datasets. Due to the relatively short length of the sequences and their high diversity, some of the major clades did not receive significant bootstrap support and were different depending on the method used, although most major clades, including Rhilphs and Shelphs, were recovered by both methods. Alphs tended to be grouped together by neighbor-joining but were split into smaller clades when maximum likelihood analysis was used. However, we still tentatively consider Alphs as a single group due to the characteristic replacements in their catalytic centre.

To circumvent the ambiguity of the results, we used Neighbor-Net [29], a neighbor-joining based method that constructs phylogenetic networks rather than trees and thus represents conflicting signals and visualises feasible trees in a single plot (Figure 4; for a high-resolution image, see Additional file 1). The Neighbor-Net analysis accurately identified the major clades such as eukaryotic and archaeal phosphatases, as well as their closer relationship to each other than to bacterial PPPs [6]. Separation of "conventional" eukaryotic PPPs into two branches, suggested previously from the analysis of the full-length catalytic domains [25], was also recovered. As it was suggested by initial sequence similarity searches, Rhilphs, Shelphs and Alphs represent distinct major clades of the PPP family, as divergent from "conventional" eukaryotic and archaeal PPP phosphatases as they are from major bacterial clades (Figure 4; Additional file 1).

### Common structural elements in all "bacterial-like" PPP phosphatases from eukaryotes and bacterial phosphatases

C-terminal regions of the catalytic domain of all "conventional" eukaryotic PPP phosphatases share a highly conserved (with minor variations) SAPNY motif, located in the β12-β13 loop. This loop and the Tyr residue of the SAPNY motif in particular are implicated in interaction with regulators and inhibitors [21,30-32]. β strands (β9 and β10) corresponding to β12 and β13 are conserved in λPP [19]. However, the sequence on the C-terminal side of β9 is dissimilar to SAPNY in λPP and in bacterial PPPs. A conservative replacement of the first Ser of SAPNY by Thr is found in many bacterial sequences (this Thr is however only moderately conserved and is replaced by Glu or Gln in all Rhilphs and by Val or Phe in most Shelphs). The two adjacent positions are occupied by highly conserved Asp and Gly residues, respectively, thus defining a previously unrecognised motif (I/L/V)D(S/T)G. This motif is present in all examined bacterial PPPs, as well as in all "bacterial-like" phosphatases from eukaryotes described above (Figure 5; see also Additional file 2 for a more complete alignment).

In addition, we note the presence of another characteristic feature of bacterial and "bacterial-like" PPPs: the His residue (H248 in PP1; H186 in λPP) coordinating one of the metal ions in the catalytic centre is preceded by an absolutely conserved Gly; this residue is conserved in some archaeal but not in "conventional" eukaryotic PPPs (Figure 5; Additional file 2).

## Discussion

**Figure 6:**
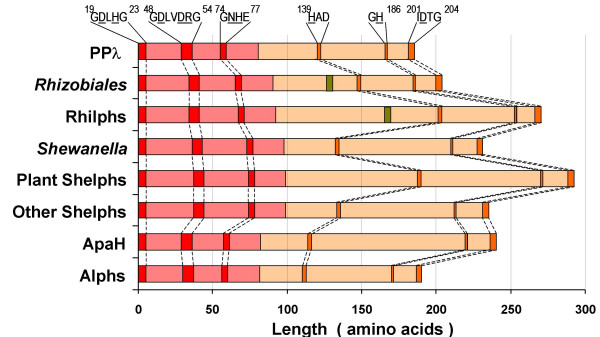
**Schematic diagram depicting organisation of the catalytic domains of the phosphatase groups discussed in this study**. N-terminal subdomains (used in the alignment for the Neighbor-Net analysis, Figure 4) and C-terminal subdomains are shown in red and yellow, respectively. Positions of the conserved motifs in PPλ and the residues forming the active centre (underlined; shown according to ref. [19]) are shown. Positions of the LXXAXPXXH motif in plant Rhilphs and related phosphatases from *Rhizobiales *are indicated (green boxes). For more detailed information on the position of inserts in Rhilphs and Shelphs relative to the elements of the secondary structure, see Figures 1 and 2, respectively.

In this report, we have documented the presence in different eukaryotic lineages of the genes that encode PPP phosphatases resembling those of bacterial origin, rather than "conventional" eukaryotic members of the family. Catalytic domains of these "bacterial-like" phosphatases are characterised by relatively conserved structure of the N-terminal subdomains, but very diverse organisation of the C-terminal subdomains, where conserved motifs and residues forming the active centre are separated by sequences of various length that share little or no similarity between different clades (Figure 6). In most cases, corresponding EST sequences could be detected, which confirms that these genes are expressed.

The most conspicuous presence of "bacterial-like" PPPs has been detected in plants. Plants possess phosphatases from all three novel groups described in this work. Rhilphs are most closely related to PPP phosphatases from a number of α-proteobacteria, including purple photosynthetic bacteria and *Rhizobiales*. The absence of related sequences in eukaryotes other than land plants suggests that Rhilphs may have been acquired after plants started colonising land. Although bacterial lineage that could be the source for plant Rhilphs could not be unambiguously identified by phylogenetic reconstruction, *Rhizobiales *(or purple photosynthetic bacteria from which *Rhizobiales *are thought to have originated [33,34]) appear to be likely candidates. Indeed, *Rhizobiales *have transmissible chromosomal elements and some, like *Agrobacterium*, are able to integrate their plasmid genes into plant genome [35] or even transform animal cells [36], a situation that would be ideally suited for a horizontal gene transfer to occur. Interestingly, the presence of genes of rhizobial origin has been detected in plant parasitic nematodes [37]. Possible origin of plant Rhilphs from α-proteobacterial phosphatases is also supported by the presence in the enzymes of both groups of characteristic inserts in similar positions, which share some sequence similarity (see Results).

Phosphatases of another group, designated as Shelphs, are found in green plants, in a red alga, in *Apicomplexa*, *Trypanosomatidae*, as well as in some fungi. The similarity between proteins from *Apicomplexa *and *Trypanosomatidae *and those from plants is well documented. *Trypanosomatidae *are related to photosynthetic euglenoids and are thought to have lost plastids secondarily [38]. Apicomplexan parasites have a relict plastid, originated from the engulfment of a red alga [39]. Thus, the presence of phosphatases shared by plants, red algae, *Apicomplexa *and *Trypanosomatidae *is not surprising and probably reflects the presence of Shelphs in a common ancestor of photosynthetic eukaryotes. The presence of chloroplast targeting sequence in SLP1 suggests a possible origin of Shelphs from a bacterial precursor of the chloroplast (however it should be noted that Shelphs are absent from cyanobacteria); alternatively, this sequence may have appeared secondarily. Protein Ser/Thr phosphorylation / dephosphorylation is essential for regulation of photosynthesis, and unidentified okadaic acid-insensitive protein phosphatases in chloroplasts have been reported [40,41]. SLP1 appears to be a good candidate for such a phosphatase.

The origin of fungal Shelphs is unclear. Curiously, they are found in basidiomycetes and in an ascomycete *S. pombe*, but not in a number of other ascomycetes, whose genomes have been completed. Current data do not permit to discriminate between (i) the presence of Shelphs in a common ancestor of eukaryotes and their loss in such lineages as animals and many fungi, and (ii) independent acquisition of Shelphs from bacteria by an ancestor of photosynthetic eukaryotes and by fungi. Further sequencing of eukaryotic genomes may shed light on the evolutionary history of this PPP group.

The third group of "bacterial-like" phosphatases detected in eukaryotes, designated here as Alphs, appears to be distantly related to bacterial diadenosine tetraphosphatases ApaH. Patchy distribution in several eukaryotic kingdoms suggests that Alphs were probably present in the common ancestor of eukaryotes, but were independently lost in many lineages, including insects, vertebrates and flowering plants. A characteristic modification of the conserved GDXXDRG motif shared only with ApaH further supports a suggestion that Alphs may represent a divergent branch of diadenosine tetraphosphatases, rather than protein phosphatases. However, relatedness of eukaryotic Alphs to bacterial diadenosine tetraphosphatases remains hypothetical, since Alph sequences are too divergent from ApaH, as well as from each other, to permit a reliable phylogenetic reconstruction.

Diadenosine oligophosphates are considered as emerging signalling molecules in both intra- and intercellular signalling in eukaryotes [42,43]. In particular, human diadenosine oligophosphate hydrolase FHIT has been identified as a tumor suppressor [44]. It seems plausible that appearance of eukaryotic diadenosine oligophosphate hydrolases (structurally unrelated to the PPP phosphatases) may have made bacterial-type diadenosine tetraphosphatases redundant, leading to their loss in many eukaryotic lineages. It would be interesting to test experimentally whether Alphs are indeed diadenosine oligophosphatases.

More generally, an important implication of our findings is that many eukaryotes possess PPP phosphatases with yet undetermined substrate specificity. Eukaryotic PPP phosphatases are generally considered as Ser/Thr specific *in vivo*, although they may be able to dephosphorylate phosphoTyr-containing substrates *in vitro *(*e.g*. [45,46]). This is probably true for archaeal PPPs as well [5]. However, Ser/Thr specificity is not a feature of bacterial PPP phosphatases [7-9,13,47-49]. Thus, it would not be possible to predict substrate specificity of uncharacterised "bacterial-like" PPP phosphatases without experimental evidence. In particular, since *Shewanella *PPI is Tyr-specific [8], it would be interesting to determine substrate specificity of eukaryotic Shelphs. It is also worth noting that interest in tyrosine phosphorylation in plants has recently been stimulated by identification of plant Tyr phosphatase genes and by the finding that Tyr phosphorylation is involved in the regulation of stomatal movement (reviewed by Luan [50]).

Three motifs, GDXHG, GDXXDRG and GNH(E/D) form the diagnostic signature of all PPP phosphatases [10,11]. We detected a (I/L/V)D(S/T)G motif, which appears to be a characteristic signature of "bacterial"-type PPPs. The existence of such a motif is striking *per se*, taking into account extreme structural diversity of bacterial PPP phosphatases. It indicates that (I/L/V)D(S/T)G was probably present as the fourth "universal" signature motif in the common ancestor of PPP phosphatases, and was lost in the common lineage of archaeal and "conventional" eukaryotic PPPs. An alternative possibility could be that the (I/L/V)D(S/T)G motif was acquired by a bacterium and propagated by lateral gene transfer, replacing the ancestral SAPNY-related motif. However this scenario seems less likely, since the (I/L/V)D(S/T)G motif is present, with minor variations, in virtually all bacterial phosphatases, despite their great diversity, and is replaced by SAPNY-related sequences only in archaeal and "conventional" eukaryotic PPPs.

The Asp residue in the 2^nd ^position of (I/L/V)D(S/T)G is highly conserved and can only be replaced by Glu, indicating that the negative charge is essential. The presence of a highly conserved Gly in the 4^th ^position indicates that flexibility of the polypeptide chain is likely to be important. The crystal structure of bacteriophage λ phosphatase (PPλ; [19]) shows that the Asp residue of (I/L/V)D(S/T)G (Asp202) is just downstream of the β9 strand, which corresponds to the β12 strand in mammalian PP1. In PPλ, Asp202 is hydrogen bonded to a water molecule coordinated to one of the metal ions in the catalytic centre, which probably accounts for its conservation. In eukaryotic or archaeal PPPs, corresponding position is occupied by neutral residues (see Figure 5).

It would be tempting to speculate that this difference in the region just downstream of the β9 (β12) may be responsible for a feature that is common to all "bacterial"-type but not to eukaryotic / archaeal PPPs. One such feature is the Ser/Thr specificity of the latter group. The Tyr residue of the SAPNY motif has been suggested to provide a bulky phenol ring in the β12-β13 loop, sufficient to sterically block access of phosphoTyr-containing substrates to the active site [32]. However this is unlikely to be the sole determinant of Ser/Thr specificity, since residues containing bulky aromatic rings (Tyr, Phe or Trp) are found in the same or adjacent positions in many bacterial phosphatases (Figure 5). Since the (I/L/V)D(S/T)G motif, absent in eukaryotic and archaeal PPPs, is involved in organisation of the catalytic centre [19], it is possible that this difference in the catalytic centre organisation may be one of the determinants of broad substrate specificity *vs*. Ser/Thr specificity.

## Conclusions

So far, eukaryotic PPP phosphatases were considered as a well-defined monophyletic group of enzymes, specifically dephosphorylating phosphoSer and phosphoThr, while a much more structurally and enzymatically diverse PPP phosphatases were known to be present in prokaryotes. Our findings demonstrate that, in addition to "conventional" eukaryotic PPP Ser/Thr-specific protein phosphatases, many eukaryotes possess very diverse "bacterial-like" PPP phosphatases. Enzymatic characteristics, physiological roles and evolutionary history of these phosphatases have yet to be revealed.

## Methods

### Detection of PPP phosphatase-coding sequences

Sequence similarity searches were conducted using BlastP or TBlastN [51] at NCBI [52] in the following databases: "non-redundant" (NR), "expressed sequence tags" (EST), "genomic sequence survey" (GSS) and "high-throughput genomic sequences" (HTGS). Additional Blast searches of the following databases were performed: finished and unfinished genomes of eukaryotes at the NCBI [53]; fungal genomes at the Broad Institute [54]; plant genomes at The Arabidopsis Information Resource (TAIR) [55]; Gene Index databases of tentative consensus sequences (EST assemblies) at The Institute for Genomic Research (TIGR) [56]; *Chlamydomonas reinhardtii *draft genome [57]. In all cases, reciprocal searches were used, i.e. hits retrieved by Blast searches were in their turn used as queries in the following Blast searches. Accuracy of gene prediction was examined by comparison of the retrieved sequences with translations of corresponding EST entries. In the absence of available ESTs, closely related sequences from other species were used. Taxonomy of the species from which the phosphatase sequences is given according to the NCBI taxonomy web site [58].

### Phylogenetic analysis

Multiple alignments were generated using CLUSTAL W [59] at Kyoto University Bioinformatics Centre [60] and edited manually. During manual editing, particular attention was paid to correct alignment of the PPP family signature motifs and other conserved residues known to constitute the catalytic site of PPP phosphatases. Phylogenetic tree construction by the neighbor-joining method [61] and bootstrap analysis were performed using the PHYLIP package, version 3.573 [26]. Maximum likelihood analysis was performed using TreePuzzle [28]. Possible alternative neighbor-joining based phylogenies were visualised using Neighbor-Net [29] as implemented in SplitsTree, version 4.β10 [62]

### Analysis of the primary structure

The presence of signal peptides and targeting sequences was analyzed using TargetP [63] at the the Centre for Biological Sequence Analysis, Technical University of Denmark [64].

## Abbreviations

EST, expressed sequence tag, Alph, ApaH – like phosphatase; PPP, protein phosphatases of the P family; Rhilph (RLP), *Rhizobiales */ *Rhodobacterales */ *Rhodospirillaceae *– like phosphatase; Shelph (SLP), *Shewanella *– like phosphatase.

## Authors' contributions

Both authors contributed equally to this work.

## Supplementary Material

Additional File 1 **Neighbor-Net analysis of the conserved N-terminal subdomains (starting 5 amino acid residues before conserved GDXHG and ending 25 residues after GNH(E/D) of 104 bacterial, archaeal and eukaryotic PPP phosphatases**. This version of Figure 4 is the original SplitsTree file that can be viewed using SplitsTree, freely available for download (see Methods). Bootstrap values (out of 100 resamplings) are shown and can be highlighted by selecting corresponding alternative splits. The file also contains the alignment used for the analysis (Input). Click here for file

Additional File 2 **Distinct conserved motifs in the C-termini of bacterial and "bacterial-like" PPP phosphatases from eukaryotes as opposed to archaeal and eukaryotic PPP phosphatases**. This is an expanded version of Figure 5. Click here for file
