# Supplementary material for: Widespread presence of "bacterial-like" PPP phosphatases in eukaryotes
Source: BMC Evol Biol. 2004 Nov 19;4:47. doi: 10.1186/1471-2148-4-47 (PMC535813; doi:10.1186/1471-2148-4-47)
Supplement: Additional File 2 — Distinct conserved motifs in the C-termini of bacterial and "bacterial-like" PPP phosphatases from eukaryotes as opposed to archaeal and eukaryotic PPP phosphatases. This is an expanded version of Figure 5. [file 1471-2148-4-47-S2.doc]

**β7** ***** **β8 β9 β10aaa**

Bacteriophage λ GADTFIF**GH**TPAVKPLKFA--------NQMY**IDTG**AVFC----GNLTLIQVQGEGA* P03772

*E. coli* GADHFWF**GH**TPLRHRVDIG--------NLHY**IDTG**AVFG----GELTLVQLQ* P55798 PrpA

*Anabaena* GVKPIIF**GH**HVVGDIPLIIA------GKIYG**IDTG**ACHG----GKLTGLILPSFEVV Q8YZT4

*Thermotoga* PGKIVVF**GH**TPFEEPFVSR--------DKIG**IDTG**CVYG----GRLTALRVEDRKFF Q9WZK1

*Fervidobacterium* LGFIVIF**GH**TPFEDVYKDA--------DKIG**IDTG**CVYG----GKLTAIELKMGTVV O34205

*Clostridium* KGYNIIC**GH**TPVQSIKNTTEVKILRRKNTFY**ID**C**G**CVYGDKAGGKLAMLRLEDFKEF Q97FF3

*Lactococcus* LNKTIVF**GH**TETKILNKNNKYDIWIHDNKIG**ID**G**G**AVYG----GYLYGVILDVHGIK Q9FB69

*Streptomyces* GGTRIVH**GH**SPIPYLLGE------VGSEDGED**DT**RTVVEG---PHVYADGLAIAMDG O87639

*Anabaena* GEALVVY**GH**TPIPEAEWLN--------NTID**IDTG**CVFG----GKLTALRYPEKELV O69213 PrpA

***Arabidopsis*** KKTIVVS**GH**HGKLHIDG----------LRLI**ID**E**G**GGYTDT---PLAAIVLPSKKII At3g09960 RLP1

***Arabidopsis*** KHTVVVS**GH**HGKLHIDG----------MRLI**ID**E**G**GGFPDK---PVAAIVLPSKKII At3g09970 RLP2

***Medicago*** SATIIVS**GH**HAKLHVEG----------LRLI**ID**E**G**GGYPDK---PIAAIVLPSMEII BQ148605

***Solanum*** TPTIIVS**GH**HAKLHIEG----------LRLV**ID**Q**G**GGFEDQ---PVAAIVLPSLEIV BQ516857

***Triticum*** KQTIVVS**GH**HGKLHVDG----------LRFI**ID**E**G**GGYADK---PIAAVVFPSKEVI BQ171163

*Sinorhizobium* WGKHVCH**GH**TPSRSNPRTVG-------NRTN**VDSG**AVFG----GMLSCAVFDDDRAG Q92V37

*Agrobacterium* LDLLVVH**GH**TPVEQPETGP--------QRIG**IDTG**AYTT----GRLTILRLSDAGLH Q8UA33

*Mesorhizobium* FPKIVVH**GH**TPVPEAEVMA--------NRVN**VDT**LAWQS----GMLTAFVVDGADKR Q987U4

*Rhodospirillum* FPHMVVH**GH**TPSREPVIRP--------HRLG**IDTG**AWTR----GMLTCVVLEGSERR ZP_00014771

*Caulobacter* LKATVVH**GH**TPVEEVFVGR--------QRIN**VDTG**AYAT----GVLTAVRLDGGEPK Q9ABQ8

***Arabidopsis*** GVKRMIM**GH**TIQDAG-INGV----CNDKAIR**ID**V**G**MSKGCADGLPEVLEIRRDSGVR At1g18480 SLP2

***Medicago*** GVKRMIM**GH**TIQKEG-INGV----CENKAIR**ID**V**G**MSKGCGGGLPEVLEIDRYGVRI AC122723

***Arabidopsis*** GAKAMVV**GH**TPQLSG-VNCE----YGCGIWR**VD**V**G**MSSGVLDSRPEVLEIRGDKARV At1g07010 SLP1

***Oryza*** GAKGMVV**GH**TPQTRG-VNSK----CDGKVWC**VD**V**G**MSYGVLYSRPEVNFRFAWKFGM Q8L676

***Porphyra***  NVKRMVV**GH**TPQMAG-ISGA----CDGRVWR**IDTG**MAKAYGG-MTKALEIDRSGKVR AV438082

***Porphyra***  GAQRMVV**GH**TIQTRG-INSA----CESRVVR**VD**V**G**MSHGCGDGPVEVLEVLKDGQVL AU192390

***Trypanosoma*** GIKSVVV**GH**VPHNHQDFKDWRL--CGGHLIA**ID**F**G**LSRWKKGDPGHVAALQIDDTTG AC007863

***Trypanosoma*** GPECVVF**GH**DARAGLQELP--------FAYG**IDTG**CVYG----GQLTAVVYGRDSPK AC084046

***Plasmodium*** SAKKLVN**GH**TIQRNRKVNEY----CKGGLIMA**DTG**ISKWKYGVINYVQYFQDGSYKV Q8I5Y5

***Plasmodium*** NAKGLVV**GH**TRQKSHEIQTY----CNNSFFL**IDTG**MSLFMNNGQPYPNYLQIEKGKF Q8IKE5

***S. pombe*** NVNRLVM**GH**TPQFHG-IVSR----CEGRILL**IDTG**LCSAYAG---ERAVLRISQNDT O74480

*Shewanella* NVNHIVV**GH**TSQERVLGL------FHNKVIA**VDS**SIKVGKS--GELLLLENNRLIRG Q9S427 PPI

*Myxococcus* GARRMVM**GH**TTNRDGKVKVR----FNGKALL**IDTG**LSTG-YGR-NLAALELRGGKVN see Table 1

***Chlamydomonas*** GVQRVVV**GH**TVQRGGRVSSR----CGGQLLM**VD**V**G**LSRAIAG---EMAVLTCSNGVL BI995255

***Pinus taeda*** MPANVVY**GH**AAARGLDINR--------WSFG**LDSG**CAYG----RKLTALVLDLPPHR CF386581

***Fragilariopsis*** GPQTVLF**GH**DADRGLQQYE--------HAIG**L**G**TG**CVYG----GRLTACILPEKRLV CF245710

***Magnaporthe*** ERMTVVY**GH**DASRGLRVPKDA---KPGNTFG**LDSG**CVYG----RELTALVIEASEKG AC127427

***Neurospora*** NRVVVVY**GH**DARSGLQVD---------KHIT**ID**P**G**KSPIPISARHITGTGDQTVIFT EAA35273

***Aspergillus*** TRTTVIF**GH**DAKRRLQLGR--------HTIG**LDS**ACLYG----HYLSALVIESTDRG AF168613

***Trypanosoma*** GPETVVF**GH**DSRRGLQEQYRP------LAIG**LDSG**CVYG----GRLSAAVFPGGHIV AC091781

***Trypanosoma*** GVKRVVV**GH**TPQRSGRVETF----CGGSVIA**ID**V**G**MSRWM---YGNIAALEITVTSY AC084046

***Leishmania*** GPEMVIF**GH**DAYAGFQAHA--------HACG**IDTG**CVYG----DPLTCVVYGQNSPA AL499620

***S. cerevisiae*** FNAKVFY**GH**DASMGLNLRR--------QTKG**LDT**ACIKN----NLLSSMKVKYDIKK P40152

***Ciona*** QGPHVYF**GH**DALRKLQTHP--------HCTG**LDTG**CVYG----GKLTAVLLKLNNFG TC9835

*E. coli* EEYSIAF**GH**WASLEGKGTPE-------GIYA**LDTG**CCWG----GTLTCLRWEDKQYF APAH_ECOL

*Ralstonia* RGTPIAF**GH**WSTRGLVMRD--------DLLG**LDTG**CVWG----GKLTAARMTLAPAG APAH_RALS

*Magnetococcus* AGEKVLY**GH**WAMAGLQLKQ--------HSLG**LDSG**CVYG----GQLTALRLDDPEHP ZP_00044402

*Thermosynecho.* DLKTIVI**GH**TITFTFPSVQAGQIVRGAGWLD**IETG**AYHQRS--GYLTALDWTNQWVY BAC09973

*Anabaena* DLKLVII**GH**TITFTFPGVNPGELAQGQGWLD**IDTG**AYHPRS--GWLTALDITNNLIY Q8YP31

*Microcystis* DFKLIIT**GH**TLTFTFPDVKPGQLARGSGWLD**IETG**VYHPQS--GWLTALDWTNQLVY O54390

*Methanosarcina* HLKRIVR**GH**TALDKGYRWWFD-----GKLLS**L**F**S**CPDYVGLGNAAAFALFEKEEIKL O34200

*Sulfolobus* NFKGIIR**GH**EAVD-GFRTNMN-----GKVIT**V**F**S**SVYHGQRSGILYHDYNNNKFVRI Q55059

*Archaeoglobus* GVKVIIRA**H**EPQK-VLKAEQD-----GRVVT**I**G**S**CALPYSISEFALLKIDFSRGFKN O28453

***H. sapiens*** NLDYIIRS**H**EVKAEGYEVAHG-----GRCVT**V**F**S**APNYCDQMGNKASYIHLQGSDLR P53041 PP5

***H. sapiens*** QLKMLIRS**H**ECKPEGYEICHD-----GKVVT**I**F**S**ASNYYEEGSNRGAYIKLCSGTTP O14829 PPEF1

***H. sapiens*** DLDLICRA**H**QVVEDGYEFFAK-----RQLVT**L**F**S**APNYCGEFDNAGAMMSVDETLMC P08129 PP1α

**β10 * β11 β12 β13**
